# Notice of Postponement

**Published:** 1875-01

**Authors:** 


*Notice of Postponement.*

We are requested by Dr. S. P. Moore, President of the Association of Medical Offieers of C. S. Army and Navy, to state that the meeting of that body appointed for July next, will be held in Richmond, Virginia, on the 19th October, 1875, at 11 o'clock A.M.

The Medical Association of Virginia convenes in Richmond on the following day—October 20th, 1875.

